# Behavioral responses and life history traits of Taiwanese and Indonesian populations of *Aedes aegypti* surviving deltamethrin–clothianidin treatment

**DOI:** 10.1186/s13071-024-06189-6

**Published:** 2024-03-07

**Authors:** Christina Natalina Silalahi, Aqsa Yasin, Mei-Er Chen, Intan Ahmad, Kok-Boon Neoh

**Affiliations:** 1 Department of Entomology, National Chung Hsing University, 145 Xingda Rd., 402 Taichung, Taiwan; 2 School of Life Sciences and Technology, Institut Teknologi Bandung, Bandung, West Java 40132 Indonesia

**Keywords:** Insecticide resistance, Hormesis, Arboviral diseases, Indoor residual spraying, Sublethal effect

## Abstract

**Background:**

Indoor residual spraying (IRS) capitalizes on the natural behavior of mosquitoes because *Aedes aegypti* commonly seeks indoor resting sites after a blood meal. This behavior allows mosquitoes to be exposed to insecticide-treated surfaces and subsequently killed. Combinations of deltamethrin and clothianidin with different modes of action have shown promise in IRS, effectively targeting both susceptible and pyrethroid-resistant malaria vectors. However, the effects of this approach on *Aedes* mosquitoes remain unclear. The present study tested the effects of deltamethrin–clothianidin mixture treatment on behavioral responses and life history traits of Taiwanese and Indonesian populations of *Ae. aegypti*.

**Methods:**

We adopted an excito-repellent approach to explore the behavioral responses of pyrethroid-resistant *Ae. aegypti* populations from Indonesia and Taiwan to a deltamethrin–clothianidin mixture used in contact irritancy and non-contact repellency treatments. We further evaluated the life history traits of surviving mosquitoes (i.e., delayed mortality after 7-day post-treatment, longevity, fecundity, and egg hatching) and investigated the potential transgenerational hormetic effects of insecticide exposure (i.e., development rate and survival of immatures and adult mosquitos).

**Results:**

All tested field populations of *Ae. aegypti* displayed strong contact irritancy responses; the percentage of escape upon insecticide exposure ranged from 38.8% to 84.7%. However, repellent effects were limited, with the escape percentage ranging from 4.3% to 48.9%. We did not observe immediate knockdown or mortality after 24 h, and less than 15% of the mosquitoes exhibited delayed mortality after a 7-day exposure period. However, the carryover effects of insecticide exposure on the survival of immature mosquitoes resulted in approximately 25% higher immature mortality than that in the control. By contrast, we further documented stimulated survivor reproduction and accelerated transgenerational immature development resulting from the sublethal effects of the insecticide mixture. In particular, the number of eggs laid by treated (both treatments) female mosquitoes increased by at least 60% compared with that of eggs laid by control female mosquitoes.

**Conclusions:**

IRS with deltamethrin–clothianidin effectively deters *Aedes* mosquitoes from entering residential areas and thereby reduces mosquito bites. However, the application rate (deltamethrin: 25 mg/m^2^; clothianidin: 200 mg/m^2^) may be insufficient to effectively kill *Aedes* mosquitoes. Insecticide response appears to vary across mosquito species; their behavioral and physiological responses to sublethal doses have crucial implications for mosquito control programs.

**Graphical Abstract:**

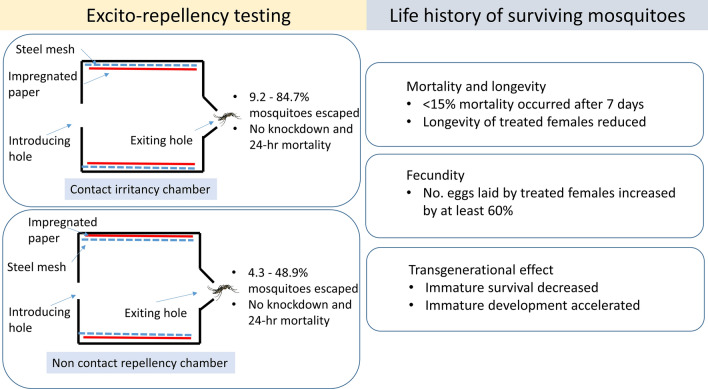

## Background

*Aedes aegypti* (Linnaeus) is a key vector of arboviral diseases, including yellow fever, chikungunya, and dengue viruses [1, 2]. No effective dengue vaccine is yet available, thus highlighting the importance of controlling *Ae. aegypti* populations and reducing dengue fever transmission. Current approaches used to combat these mosquitoes are primarily focused on integrated vector management, encompassing biological, mechanical, and chemical methods [3–7].

Chemical control remains a vital measure for managing anthropophilic disease vectors during outbreaks [8, 9]. Various chemical control methods, including the space spraying of insecticides and the application of larvicides, have limitations in effectively suppressing mosquito populations [9]. For instance, space spraying has a short residual effect, necessitating continuous application for successful mosquito control [10, 11]. Moreover, adult *Ae. aegypti* mosquitoes typically seek indoor resting sites and hide in cryptic places (e.g., at a height of < 1.5 m, especially in dark areas, under beds, beneath household furniture, or on hanging clothes), with their primary food source being human blood [12, 13]. This behavior makes outdoor spraying methods, such as fogging or ultra-low volume fogging, less effective in controlling the spread of dengue fever [14]. Therefore, alternative control strategies to complement traditional approaches are urgently required.

Indoor residual spraying (IRS) has emerged as an effective method for reducing *Ae. aegypti* populations and preventing dengue transmission [3, 15–17]. IRS leverages the natural behavior of *Ae. aegypti*, which typically rests indoors after feeding on blood. This behavior exposes mosquitoes to insecticide-treated surfaces, eventually killing them [13, 17, 18].

An insecticide mixture containing clothianidin and deltamethrin complements the existing pyrethroid (deltamethrin)-based IRS. Deltamethrin acts on nerve membranes by delaying the closure of the activation gate for the sodium ion channel. Clothianidin functions as a neonicotinoid insecticide agonist, targeting the nicotinic acetylcholine receptor in the insect central nervous system. The combination of these two active ingredients with different modes of action has shown promise in IRS, effectively targeting malaria vectors both susceptible and resistant to pyrethroid [19–23]. Studies evaluating the insecticidal activity of IRS have often used standard World Health Organization cone bioassays [24]. However, some controversy exists surrounding the use of these bioassays to quantify residual spraying efficacy, because they may not fully represent natural mosquito behavior. Deltamethrin, a neurotoxin, can induce contact irritancy in mosquitoes, prompting avoidance behavior and escape from treated areas within a short time [25, 26]. Yu et al. [27] revealed that at least 3.6–31.2% of field *Ae. aegypti* mosquitoes exhibited avoidance behavior and escaped deltamethrin-treated areas within 30 min. Furthermore, Kongmee et al. [28] reported that six field populations of *Ae. aegypti* exhibited strong escape responses (32–78%) within 30 min of contact with deltamethrin. This escaping behavior may affect the mortality of mosquitoes exposed to insecticide-treated surfaces.

Resistant mosquitoes often exhibit delayed mortality. For example, Fongnikin et al. [22] observed that pyrethroid-resistant *Anopheles gambiae* (sensu lato) exposed to Fludora Fusion (a mixture of deltamethrin and clothianidin) exhibited elevated mortality rates, increasing from approximately 20% after 24 h to 70% after 120 h of exposure, with 30% of all mosquitoes surviving at the end of the experiment. These survivors can mate with individuals of the opposite sex and reproduce, thus contributing to population persistence. However, in most IRS trials, mortality was recorded for a duration of only up to 72 or 96 h, and the life history of exposed mosquitoes beyond the experimental period was not considered [19, 20, 23].

A growing body of evidence suggests that sublethal or low doses of pyrethroids and neonicotinoids affect mosquito physiology and vital behaviors, including host-seeking. For instance, Cohnstaedt and Allan [29] reported significant reductions in activation time to flight and flight direction in *Ae. aegypti* mosquitoes that survived deltamethrin–permethrin treatment. Rigby et al. [30] demonstrated that exposure to sublethal permethrin doses reduced egg viability, blood avidity, male mating success, and longevity in susceptible *Ae. aegypti*; this treatment also reduced host-location success by 20–30% in *Ae. aegypti* [30]. However, insecticide-resistant female mosquitoes exhibited increased reproductive output and mating success rates after exposure, which indicates that exposure to sublethal pyrethroid doses is not necessarily detrimental to mosquitoes [30]. The phenomenon of low-dose stimulation response (also known as hormesis), where exposure to a sublethal insecticide dose exerts a stimulatory effect on certain aspects of the insect’s physiology, has been observed in various insect species [31]. However, the effects of sublethal neonicotinoid doses on the fitness of *Aedes* mosquitoes remain poorly studied. Therefore, investigating the life history traits of survivors and their offspring is imperative.

In this study, we adopted the excito-repellent (ER) approach to examine the behavioral responses of pyrethroid-resistant *Ae. aegypti* populations from Indonesia and Taiwan when exposed to a mixture of deltamethrin and clothianidin. The ER approach enabled us to investigate both the voluntary escape behavior triggered by insecticidal contact irritancy (the mosquitoes may be irritated when contacting a treated surface) and non-contact repellency (the mosquito may be repelled by the presence of insecticide before contacting a treated surface), thus facilitating a comprehensive assessment of the insecticide’s effectiveness. In addition, we investigated the life history traits of surviving mosquitoes and the potential transgenerational hormetic effects of exposure to the insecticide mixture.

## Methods

### Mosquito sampling

We collected 18 populations of *Ae. aegypti* from Indonesia and Taiwan. In Indonesia, dengue fever caused by dengue virus is a widespread vector-borne disease [32], affecting approximately 93.58% of Indonesia’s regencies/cities in 2019 [33]. By contrast, dengue is not endemic to Taiwan, but sporadic local outbreaks occur due to an increasing number of local travelers visiting dengue-endemic countries. Thermal fogging with pyrethroid insecticides is used as an immediate remedial control measure when the mosquito density is above a certain threshold or a case of dengue fever is reported.

We randomly selected 11 *Ae. aegypti* populations from regencies/cities located in 11 Indonesian provinces where dengue cases had been reported [34]. Between May and December 2019, *Ae. aegypti* eggs were obtained using ovitraps from the following areas in Indonesia: Sumatera, Medan, North Sumatra (3°33′20.8″N, 98°37′45.6″E); Pangkal Pinang, Bangka Belitung (2°07′13.3″S, 106°06′17.6″E); Batam, Kepulauan Riau (1°06′20.9″N, 103°57′48.0″E); Java, Kelapa Gading, DKI Jakarta (6°8′53.8″S, 106°54′29.4″E); Semarang, Central Java (6°59′30.7″S,110°25′00.7″E); Kiaracondong, Bandung, West Java (6°55′48.5″S, 107°39′23.8″E); Kalimantan, Kapuas, Central Kalimantan (3°14′40.5″S, 114°23′55.91″E); Samarinda, East Kalimantan (0°28′13.39″S, 117°9′11.13″E); Pontianak, West Kalimantan (0°03′32.1″S, 109°19′44.3″E); Sulawesi, Makassar, South Sulawesi (5°08′35.2″S, 119°26′39.4″E); and Papua, Jayapura, Papua (2°34′12.2″S, 140°41′55.4″E). In addition, seven *Ae. aegypti* populations were collected from the following areas in southern Taiwan: Tainan, Zhongxi District (22°59′47.3″N, 120°11′38″E); North District (23°00′30.4″N, 120°12′27.7″E); Annan District (23°03′30.6″N, 120°08′08.7″E); Kaohsiung, Sanmin District (22°38′53.9″N, 120°19′35.5″E); Xiaogang District (22°33′18.4″N, 120°21′49.4″E); Lingya District (22°37′24.9″N, 120°19′1.3″E); and Qianzhen District (22°36′2.23″N, 120°18′53.23″E). All 18 populations have been reported to exhibit low to high levels of resistance to deltamethrin [27, 35]

### Mosquito rearing

Eggs collected from each location were hatched in plastic containers (29.5 cm × 23.0 cm × 5.0 cm) filled with 1 L of dechlorinated water. The larvae were fed an artificial larval diet consisting of pork liver powder and yeast at a ratio of 1:1. Emerged *Ae. aegypti* adults were fed with 10% sucrose solution and maintained at a temperature of 25 °C ± 1 °C and a relative humidity of 65 ± 5% under a constant 12-h light/dark photoperiod. Female mosquitoes were provided with pig blood using an artificial feeder device to promote reproduction. Adult *Ae. aegypti* progenies up to generation F5 were used in the bioassays. A susceptible reference strain, the Bora Bora strain (F35), was used for comparison.

### Evaluation of behavioral responses of field *Aedes* mosquitoes

The ER test was conducted to examine the behavioral avoidance (repellency and contact irritancy) [36] of *Ae. aegypti* toward Fludora Fusion (an insecticide containing 500 g/kg of clothianidin and 62.5 g/kg of deltamethrin). The ER test apparatus comprised four chambers, each containing two treatment and control chambers. In one treatment chamber, the internal lining featured insecticide-impregnated paper to allow mosquitoes to make physical contact (referred to as "contact"); The other treatment chamber had mesh placed in a position to prevent mosquitoes from making physical contact with the test paper surfaces (referred to as "non-contact"). The filter paper (14 cm × 17 cm) was impregnated with the recommended dosages of insecticides (deltamethrin: 25 mg/m^2^; clothianidin: 200 mg/m^2^). In the control group, the filter paper was treated with water [36].

To standardize their physiological condition, female mosquitoes were starved of sucrose solution and blood meal for 12 h before analyses. Fifteen 5- to 7-day-old unmated female *Ae. aegypti* were introduced into each chamber. Before the observation, mosquitoes were allowed to acclimatize for 3 min in the test chamber. The number of mosquitoes escaping from each chamber to the receiving box through an exit hole (designated as escaped mosquitoes) was recorded every minute for 30 min. In addition, the number of mosquitoes remaining inside each chamber (designated as non-escaped mosquitoes) was recorded after the ER bioassay. The experiment was conducted under the aforementioned laboratory conditions and replicated four times for each population.

### Knockdown and mortality

All mosquitoes that escaped or remained inside the test chambers were transferred to respective paper cups (measuring 7.5 cm in diameter and 9.5 cm in height) and provided with 10% sucrose solution. The rate of mortality was determined 24 h after exposure.

### Evaluation of post-treatment life history traits

For this study, mosquito populations from the Sanmin, North, and Zhongxi districts in Taiwan were used. Non-escaped survivors from both contact irritancy and spatial repellency treatments were placed in a paper cup and provided with 10% sucrose solution. Each paper cup contained a plastic cup (measuring 4.0 cm in diameter and 2.5 cm in height), which was filled with water and lined with filter paper (13.0 cm in length and 2.0 cm in width) to make it an oviposition site [37]. Each female was paired with a male from the same population. After 7 days of pairing, eggs laid by each female were collected, counted, and dried for further analysis. Blood meals were provided on a weekly basis until all female mosquitoes had died. The numbers of deaths and eggs laid were recorded daily. The experiment was replicated four times for each population.

### Egg hatchability assessment

To assess egg hatchability, the batch of eggs laid by each female mosquito was transferred to a 10-cm plastic cup filled with dechlorinated water. The number of eggs that hatched was recorded daily for 3 days.

### Development of immature mosquitoes

Newly emerged first-instar larvae were transferred to plastic containers filled with 1 L of dechlorinated water. The larvae were provided with 0.5 g of an artificial diet (ad libitum) containing dried pork liver powder and dried yeast at a ratio of 1:1. The numbers of larvae, pupae, and adult mosquitoes were recorded daily.

### Wing length measurement

Newly emerged female mosquitoes were transferred to mosquito cages (measuring 32.5 cm in length, 32.5 cm in width, and 32.5 cm in height) and provided with 10% sucrose solution. Subsequently, 30 5-day-old female mosquitoes from each population were randomly collected and stored at −20°C for wing length measurement. A pair of wings from each female was mounted on a microscope slide (Leica S8 AP0, Leica Microsystems, Heerbrugg, Switzerland). A digital image of the wings was captured using a camera (EI200 HD, OPTI Advanced Imaging Ltd., Taipei City, Taiwan), and the distance between veins R3 and R4+5 was measured from the axillary incision (or alula notch) to the apex of the wing, excluding the fringe scales [37]. The images were analyzed using Helicon Focus Lite version 6.7.1 (Helicon Soft Ltd., Kharkiv, Ukraine).

### Data analysis

The observed mean escape percentage for the 30-min exposure was adjusted using the Abbot formula, considering the control mosquitoes. The Kolmogorov–Smirnov *Z*-test was performed to examine the normal distribution of the data. Data regarding the rates of mosquito escape, knockdown, mortality, egg hatching, and immature survival were subjected to arcsine square root transformation. In addition, data on egg numbers were subjected to log_10_ transformation. A one-way analysis of variance test, followed by Tukey’s honestly significant difference test, was conducted to compare the mean percentages of escape, knockdown, and mortality among the field populations of *Ae. aegypti* for both contact irritancy and non-contact repellency treatments. In addition, a probit analysis was performed to calculate the escape time of 50% (ET_50_) of all mosquitoes in each population. An independent-samples *t*-test was performed to determine differences in the life history of mosquitoes between the treatment and control groups. Kaplan–Meier survival analysis was performed to estimate the mean lifespan of treated and control female mosquitoes. The log-rank test was used to compare lifespan between treated and control female mosquitoes. Statistical significance for all tests was set at *P* < 0.05.

## Results

### Behavioral response

The escape percentage was higher in the contact irritancy treatment (9.2–84.7%) than in the non-contact repellency treatment (4.3–48.9%) (Tables 1 and 2). Significant differences in the escape percentage were observed between the populations exposed to a deltamethrin–clothianidin mixture in the contact irritancy treatment (*F*_(18,57)_ = 5.057, *P* < 0.05). In most field populations from Indonesia, the escape percentage after 30 min of exposure ranged from 48.6% to 84.7%, and these values were statistically similar to the escape percentage of the Bora Bora strain (69.0 ± 7.0%; Table 1). However, the field population from Kapuas, which exhibited high resistance to deltamethrin, had a significantly lower escape percentage (9.2 ± 1.8%; Table 1) than the Bora Bora strain. The mean escape time for the Bora Bora strain was 4.81 min (% fiducial limit: 3.3–6.2 min). Out of 11 field mosquito populations from Indonesia, four required < 7 min to escape the treatment chamber; this value did not significantly differ from that of the Bora Bora strain. However, the field populations from Kapuas, Samarinda, and Jayapura exhibited relatively non-responsive behaviors to the insecticide mixture, requiring > 25 min to escape from the treatment chamber (Table 3). Among the seven field strains collected from Taiwan, the Qianzhen population, which showed the highest level of resistance to deltamethrin, exhibited the lowest escape percentage, with 24.1 ± 10.0% escaping within 30 min of exposure. The other six field strains exhibited escape percentages ranging from 38.8% to 75.8% after 30 min of direct contact with treated surfaces (Table 1). All populations from Taiwan required significantly more time to escape from the treatment chambers (ranging from 12 to 48 min) than did the Bora Bora strain, except for Zhongxi, which escaped within 5 min (Table 3).

**Table 1:** Escape and mortality rates of *Aedes aegypti* exposed to deltamethrin–clothianidin in the contact irritancy treatment

Test populations	RR50 to deltamethrin	Mean of escaping mosquito ± SE (%)	Percent knockdown (Kd) and mortality in treatments			
			% Kd 30-min exposure		% Mortality 24 h	
			Esc ± SE	Non-esc ± SE	Esc ± SE	Non-esc ± SE
*Laboratory strain*						
Bora Bora		69.0 ± 7.0^ab^	0	27.28 ± 7.23	22.92 ± 14.60	27.28 ± 7.23
*Indonesia population*						
Kiaracondong	7.45	68.5 ± 2.1^ab^	0	0	0	0
Batam	8.69	80.0 ± 7.1^a^	0	0	0	0
Jayapura	9.73	50.2 ± 12.4^abc^	0	0	0	0
Semarang	10.98	71.4 ± 8.9^ab^	0	0	0	0
Belitung	11.95	84.7 ± 2.8^a^	0	0	0	0
Makassar	12.72	67.9 ± 4.1^ab^	0	0	0	0
Pontianak	20.37	69.6 ± 8.0^ab^	0	0	0	0
Medan	22.62	77.0 ± 15.8^a^	0	0	0	0
Gading	24.35	82.8 ± 8.9^a^	0	0	0	0
Samarinda	30.76	48.6 ± 6.9^abc^	0	0	0	0
Kapuas	35.29	9.2 ± 1.8^c^	0	0	0	0
*Taiwan population*						
Annan	1.73	42.7 ± 11.0^abc^	0	0	0	0
Zhongxi	1.76	75.8 ± 6.6^a^	0	0	0	0
Sanmin	2.13	45.0 ± 6.2^abc^	0	0	0	0
North	2.17	66.2 ± 16.9^ab^	0	0	0	0
Xiaogang	2.69	56.0 ± 15.1^abc^	0	0	0	0
Lingya	2.79	38.8 ± 3.2^abc^	0	0	0	0
Qianzhen	88.64	24.1 ± 10.0^bc^	0	0	0	0

*RR50* resistance ratio, *SE* standard error, *Esc* escaped, *Non-esc* non-escaped

The values of RR50 were adopted from studies conducted by Yu et al. [27] and Silalahi et al. [35]. The mean escape percentage was adjusted considering the control mosquitoes using the Abbot formula. Numbers followed by different letters indicate significant differences between the field populations of *Aedes aegypti* (*P* < 0.05, analysis of variance followed by Tukey’s honestly significant difference test)

**Table 2:** Escape and mortality percentages of *Aedes aegypti* exposed to deltamethrin–clothianidin in the non-contact repellency treatment

Test populations	RR50 to deltamethrin	Mean of escaping mosquito ± SE (%)	Percent knockdown (Kd) and mortality in treatments			
			% Kd 30-min exposure		% Mortality 24 h	
			Esc ± SE	Non-esc ± SE	Esc ± SE	Non-esc ± SE
*Laboratory strain*						
Bora Bora		4.2 ± 4.2^a^	0	0	0	0
*Indonesia population*						
Kiaracondong	7.45	8.1 ± 5.3^ab^	0	0	0	0
Batam	8.69	21.8 ± 11.5^ab^	0	0	0	0
Jayapura	9.73	17.4 ± 1.8^ab^	0	0	0	0
Semarang	10.98	10.5 ± 3.3^ab^	0	0	0	5.13 ± 5.13
Belitung	11.95	13.7 ± 7.5^ab^	0	0	0	0
Makassar	12.72	28.9 ± 14.8^ab^	0	0	0	0
Pontianak	20.37	27.6 ± 16.0^ab^	0	0	2.38 ± 2.38	0
Medan	22.62	33.3 ± 1.5^ab^	0	2.22 ± 2.22	0	2.22 ± 2.22
Gading	24.35	34.8 ± 7.2^ab^	0	0	0	0
Samarinda	30.76	19.3 ± 9.8^ab^	0	0	0	0
Kapuas	35.29	5.3 ± 4.0^ab^	0	0	0	0
*Taiwan population*						
Annan	1.73	18.5 ± 10.0^ab^	0	0	0	0
Zhongxi	1.76	48.9 ± 9.1^b^	0	0	0	0
Sanmin	2.13	11.5 ± 2.4^ab^	0	0	0	0
North	2.17	39.5 ± 10.6^ab^	0	0	0	0
Xiaogang	2.69	15.3 ± 2.7^ab^	0	0	0	0
Lingya	2.79	4.3 ± 4.3^a^	0	0	0	0
Qianzhen	88.64	19.2 ± 9.7^ab^	0	0	0	0

*RR50* resistance ratio, *SE* standard error, *Esc* escaped, *Non-esc* Non-escaped

The values of RR50 were adopted from studies conducted by Yu et al. [27] and Silalahi et al. [35]. The mean escape percentage was adjusted considering the control mosquitoes using the Abbot formula. Numbers followed by different letters indicate significant differences between the field populations of *Aedes aegypti* (*P* < 0.05, analysis of variance followed by Tukey’s honestly significant difference test)

**Table 3:** Time required for 50% (ET_50_) of mosquitoes (belonging to different *Aedes aegypti* populations) to escape treatment chambers

Test populations	Contact (min) (95% fiducial limit)	Non-contact (min) (95% fiducial limit)
*Laboratory strain*		
Bora Bora	4.81 (3.33–6.21)	na
*Indonesia population*		
Kiaracondong	7.59 (6.41–8.76)	na
Batam	6.97 (5.92–8.01)	82.63 (55.09–165.01)
Jayapura	25.47 (20.98–33.31)	na
Semarang	7.25 (6.45–8.03)	39.53 (27.98–72.74)
Belitung	5.04 (4.44–5.62)	38.69 (30.59–54.66)
Makassar	11.31 (9.94–12.78)	28.95 (24.32–37.10)
Pontianak	10.99 (9.81–12.22)	39.53 (27.98–72.74)
Medan	2.70 (1.62–3.77)	30.40 (24.31–41.90)
Gading	5.29 (4.42–6.12)	18.28 (15.20–22.88)
Samarinda	26.02 (22.47–31.42)	90.23 (55.87–235.01)
Kapuas	71.61 (49.85–129.28)	na
*Taiwan population*		
Annan	14.12 (12.02–16.71)	46.16 (35.86–67.72)
Zhongxi	5.07 (4.14–5.96)	37.28 (25.95–75.56)
Sanmin	23.38 (20.17–28.16)	na
North	11.75 (10.43–13.15)	26.12 (21.36–34.64)
Xiaogang	15.81 (13.21–19.32)	na
Lingya	47.86 (33.61–86.77)	na
Qianzhen	27.63 (21.33–41.56)	26.89 (21.39–37.45)

*na* not applicable because few mosquitoes escaped from the chambers

A post hoc analysis of the results of the non-contact repellency treatment revealed that only the escape percentage of the Zhongxi population (48.9 ± 9.1%) varied significantly from that of the Bora Bora strain (4.2 ± 4.2%; *F*_(18,57)_ = 2.281, *P* < 0.05; Table 2). The non-responsiveness of mosquitoes in the non-contact repellency treatment was supported by their delayed escape times, with most field mosquito populations requiring at least 26 min (ET_50_) to escape from the treatment chamber (Table 3).

### Knockdown and mortality

In the contact irritancy treatment, the susceptible Bora Bora strain exhibited a knockdown percentage of 27.3 ± 7.2% and 24-h mortality of 27.3 ± 7.2% when exposed to the insecticide mixture for 30 min. No knockdown was observed for mosquitoes that escaped from the treatment chamber during the 30-min exposure period. However, subsequent mortality of 22.9 ± 14.6% was noted among the escaped mosquitoes at 24 h post-exposure. No knockdown or 24-h mortality was recorded in the escaped or non-escaped mosquitoes for any of the field populations of *Ae. aegypti* (Table 1). In the non-contact repellency treatment, both the susceptible Bora Bora strain and field populations exhibited negligible knockdown and mortality, regardless of whether mosquitoes escaped from the treatment chamber (Table 2).

### Delayed mortality and longevity of survivors

On day 7 after exposure to the insecticide mixture, the rate of mortality was significantly higher among female mosquitoes from North (10.1 ± 1.7%) and Zhongxi (11.4 ± 2.3%) than among control female mosquitoes (North: 1.6 ± 1.6%; Zhongxi: 0.0 ± 0.0%) (Table 4). However, no significant difference in delayed mortality was observed between treated and control female mosquitoes from the Sanmin population in the contact irritancy treatment (Table 4). In the non-contact repellency treatment, only the population from North exhibited a significant difference in delayed mortality between treated female mosquitoes (15.5 ± 1.5%) and control female mosquitoes (North: 8.8 ± 1.5%) (Table 5). The Kaplan–Meier survival analysis revealed that the lifespan of treated female mosquitoes was significantly shorter than that of control mosquitoes in both contact irritancy (except for Sanmin and North populations) and non-contact repellency treatments (Table 4 and 5).

**Table 4:** Biological attributes (mean ± standard error) of control *Aedes aegypti* field populations and mosquitoes surviving the deltamethrin–clothianidin contact irritancy treatment

	North	Sanmin	Zhongxi
*Mortality on day 7 (%)*			
Control	1.6 ± 1.6	1.6 ± 1.6	0.0 ± 0.0
Exposed	10.1 ± 1.7	6.9 ± 2.7	11.4 ± 2.3
*P*-value	0.01	0.15	0.01
*Longevity (day)*			
Control	19 (16–21)	20 (18–23)	21 (19–24)
Exposed	16 (14–18)	18 (16–21)	18 (16–21)
* P*-value	0.06	0.16	0.02
*Mean eggs/female*			
Control	29 ± 3	27 ± 4	51 ± 5
Exposed	46 ± 11	62 ± 9	86 ± 5
*P*-value	0.19	0.15	0.01
*Egg hatching (%)*			
Control	72.2 ± 5.3	71.8 ± 1.5	73.0 ± 4.7
Exposed	71.0 ± 5.4	72.4 ± 1.4	71.0 ± 1.0
*P*-value	0.89	0.77	0.69
*Immature survival (%)*			
Control	88.2 ± 3.6	86.1 ± 1.7	89.6 ± 3.0
Exposed	75.3 ± 1.3	73.8 ± 3.5	75.8 ± 4.6
*P*-value	0.02	0.02	0.04
*Wing length (mm)*			
Control	3.51 ± 0.05	3.47 ± 0.04	3.48 ± 0.01
Exposed	3.50 ± 0.03	3.47 ± 0.04	3.48 ± 0.01
*P*-value	0.69	0.43	0.95

**Table 5:** Biological attributes (mean ± standard error) of control *Aedes aegypti* field populations and mosquitoes surviving the deltamethrin–clothianidin non-contact repellency treatment

	North	Sanmin	Zhongxi
*Mortality on day 7 (%)*			
Control	8.8 ± 1.5	0.0 ± 0.0	1.6 ± 1.6
Exposed	15.5 ± 1.5	6.9 ± 0.2	5.7 ± 1.9
*P*-value	0.02	0.15	0.15
*Longevity (day)*			
Control	17 (14–19)	20 (17–23)	22 (19–21)
Exposed	15 (13–16)	16 (14–18)	19 (19–24)
*P*-value	0.04	0.01	0.02
*Mean eggs/female*			
Control	17 ± 2	32 ± 5	45 ± 8
Exposed	28 ± 5	53 ± 7	77 ± 9
*P*-value	0.14	0.06	0.04
*Egg hatching (%)*			
Control	62.0 ± 0.2	73.2 ± 4.0	73.2 ± 1.4
Exposed	66.7 ± 3.8	64.0 ± 0.6	70.1 ± 0.3
*P*-value	0.36	0.12	0.12
*Immature survival (%)*			
Control	90.8 ± 1.2	94.0 ± 1.9	87.7 ± 3.5
Exposed	92.6 ± 2.7	96.0 ± 1.1	76.1 ± 2.3
*P*-value	0.54	0.48	0.03
*Wing length (mm)*			
Control	3.51 ± 0.01	3.49 ± 0.01	3.49 ± 0.03
Exposed	3.51 ± 0.01	3.49 ± 0.01	3.49 ± 0.01
*P*-value	0.70	0.67	0.22

### Fecundity

Regarding fecundity, in the contact irritancy treatment, the mean number of eggs laid by female mosquitoes from the North, Sanmin, and Zhongxi populations increased by 59%, 130%, and 68%, respectively, compared with the respective control groups (Table 4). However, only the increase in the Zhongxi population was statistically significant (Table 4). In the non-contact repellency treatment, the mean number of eggs laid by female mosquitoes from the North, Sanmin, and Zhongxi populations increased by 65%, 66%, and 71%, respectively, compared with the respective control groups (Table 5). However, no significant difference was noted between treated and control mosquitoes in the North and Sanmin populations (Table 5).

### Egg hatching

**Fig. 1:**
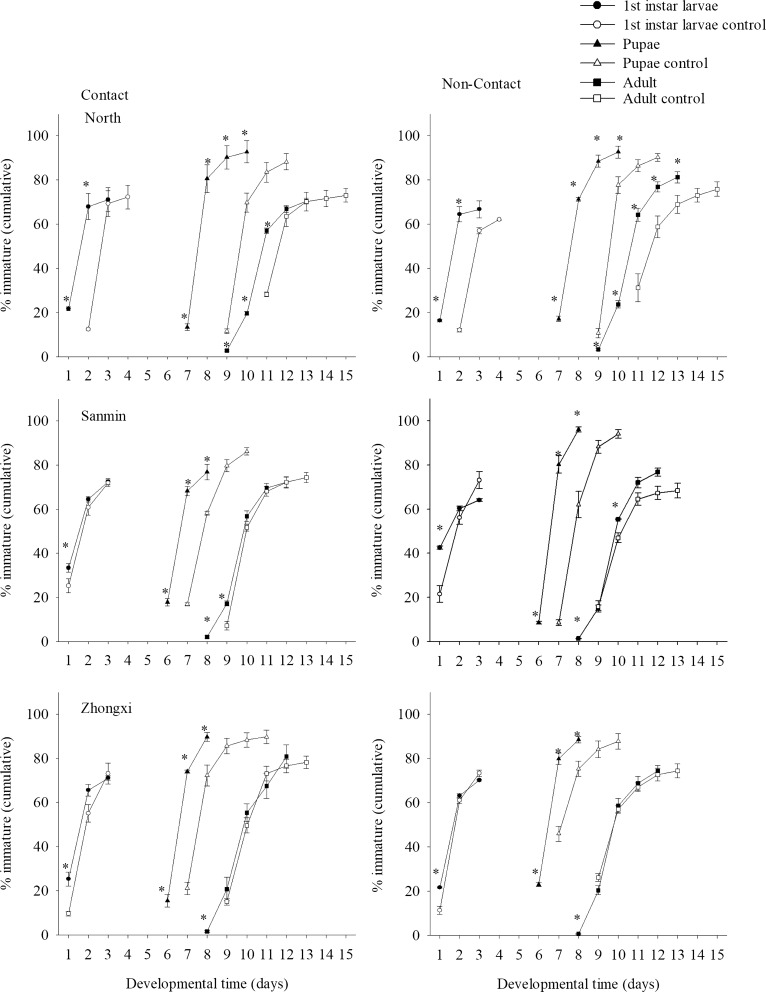
Rates of the hatching of eggs and the development of larvae and pupae produced by female mosquitoes exposed to the deltamethrin–clothianidin mixture (contact) and spatially repelled by the deltamethrin–clothianidin mixture (non-contact). * Indicates that the proportions of progenies produced by treated female mosquitoes were significantly higher than those of progenies produced by control female mosquitoes

The rates of egg hatching did not vary significantly between control and treated female mosquitoes in the contact irritancy (Table 4) or non-contact repellency treatment groups (Table 5). However, in the contact irritancy treatment, eggs laid by treated female mosquitoes from the North population hatched 1 day earlier than those laid by control female mosquitoes. For treated mosquitoes, the rates of egg hatching were 67.9 ± 5.8% and 64.4 ± 3.4% on day 2 in the contact irritancy and non-contact repellency treatments, respectively (Fig. 1). No significant difference in the hatching rate was observed between eggs laid by treated female mosquitoes from the Sanmin and Zhongxi populations in either treatment (Fig. 1).

### Survival and development of immature mosquitoes

In terms of immature survivorship, the proportion of immature mosquitoes that successfully transformed into adults in the progeny produced by female mosquitoes subjected to the contact irritancy treatment was significantly lower than those produced by control female mosquitoes (Table 4). In the non-contact repellency treatment, a significant reduction was observed in immature survivorship in the progeny produced by treated female mosquitoes from the Zhongxi population (Table 5).

The development of larvae and pupae produced by female mosquitoes that came into contact with the insecticide mixture was significantly accelerated (Fig. 1). Similarly, larvae and pupae produced by female mosquitoes in the non-contact repellency treatment exhibited a significantly increased rate of development into adults compared with that noted in the progeny produced by control female mosquitoes (Fig. 1).

### Wing length

No significant difference in wing length was observed between the progenies produced by treated and control female mosquitoes in either treatment (Table 4 and 5).

## Discussion

Insecticide resistance has been reported in *Ae. aegypti* populations in Indonesia [35] and Taiwan [27, 38], underscoring the need for strategies for managing insecticide resistance, including the use of mixtures of insecticides with different modes of action. An alternative strategy involves IRS with insecticides, which capitalizes on mosquitoes’ tendency to seek indoor resting sites after a blood meal, thereby enhancing the likelihood of contact with the insecticide. Our study results demonstrated that all the tested field populations of *Ae. aegypti* displayed a strong contact irritancy response upon exposure to the deltamethrin–clothianidin mixture, whereas repellent effects were limited. Although we did not observe immediate knockdown or mortality, we detected significant but < 10% delayed mortality 7 days after treatment in the North populations. In addition, exposure to the insecticide mixture affected the life history traits of exposed female mosquitoes and the survival and development of their offspring.

The behavioral response of *Ae. aegypti* from Thailand and Taiwan to deltamethrin has been associated with insecticide resistance and cuticular thickness in field mosquitoes [27, 39]. Resistant field populations typically exhibited a limited response to deltamethrin-treated surfaces, with < 35% of field mosquitoes escaping from such surfaces, compared with the approximately 75% escape observed in laboratory-susceptible mosquitoes [27]. However, in our study, regardless of the insecticide resistance status, all tested *Ae. aegypti* populations exhibited a high escape rate (ranging from 40 to 80%) upon contact with the insecticide mixture-treated surfaces. This finding indicated that the contact irritancy response of field *Ae. aegypti* to the insecticide mixture was stronger than that to deltamethrin alone. This finding aligns with the results reported by Fongnikin et al. [22], who investigated the effect of this insecticide mixture by applying the hut trial procedure to a wild, free-flying pyrethroid-resistant *An. gambiae* (s.l.) population from Cové, Benin. The researchers reported that exposure to the insecticide mixture led to significantly higher levels of early mosquito exiting (55–60%) than did exposure to clothianidin alone (37–38%) [22]. Moreover, the results suggest that the insecticide mixture can keep female mosquitoes out of treated houses, leading to a reduction in mosquito bites on humans.

The early escape of mosquitoes from treated surfaces may lead to reduced contact time with the insecticide, resulting in reduced insecticide intake and increased survival rates after treatment. A study demonstrated that field *Ae. aegypti* mosquitoes from Taiwan and Thailand exhibited negligible mortality rates upon exposure to deltamethrin (application rate: 25 mg active ingredient (a.i.)/m^2^) alone in contact irritancy (< 11%) and non-contact repellency (< 6%) treatments [27]; even the addition of clothianidin did not enhance the 24-h mortality rate in the present study. Our results indicated that neither knockdown nor mortality occurred in either treatment, regardless of whether the mosquitoes escaped from the test chambers. However, we observed that adult female mosquitoes exposed to the insecticide mixture exerted sublethal carryover effects on immature mosquitoes in the contact irritancy treatment, resulting in an immature mortality rate of approximately 25%.

In the present study, we observed limited delayed mortality; the mortality of female mosquitoes significantly increased to 10% after 7 days of post-exposure compared with the rate noted in the control group from North populations. This finding differs from that of the hut trial, where the mortality of pyrethroid-resistant *An. gambiae* increased from approximately 25% after 24 h to 69% after 120 h [22]. Despite methodological differences, the mortality of *Aedes* mosquitoes in this study was considerably lower than that of *Anopheles* mosquitoes [22]. The lower mortality of *Aedes* mosquitoes can be attributed to several factors, including differences in insecticide susceptibility between *Aedes* and *Anopheles* mosquitoes. For example, the LD_50_ of deltamethrin for susceptible *Ae. aegypti* larvae is substantially higher (0.770 mg/l) than that for susceptible *An. gambiae* larvae (0.0068 mg/l) [40, 41]. The LD_50_ of imidacloprid (a neonicotinoid) for susceptible *Ae. aegypti* adults (7.7 × 10^−4^ μg/mg) is higher than that for susceptible *Anopheles quadrimaculatus* (3.8 × 10^−4^ μg/mg) [42]. This finding suggests that a higher insecticide dose is required to ensure similar levels of mortality in *Ae. aegypti* and *Anopheles* mosquitoes. Overall, the discrepancy in mortality rates between *Aedes* and *Anopheles* mosquitoes highlights the complexity of insecticide responses across mosquito species and warrants further investigation to fully elucidate the underlying mechanisms. Moreover, it underscores the importance of carefully considering the appropriate application rate and insecticide mixtures when designing mosquito control strategies to effectively combat insecticide-resistant *Aedes* mosquitoes.

We observed that the lifespan of treated female mosquitoes was significantly reduced by 2 to 4 days compared with that of control female mosquitoes after both contact irritancy and non-contact repellency treatments. The survivors remained reproductively active and could mate with male mosquitoes. We initially anticipated that although female mosquitoes survived the treatment, their reproductive output would be significantly reduced due to the sublethal effects of deltamethrin and clothianidin, as observed in other insects [43–46]. However, we found that the mean number of eggs produced per surviving female increased by at least 60% compared with that produced by control mosquitoes. This unexpected finding suggests a low-dose stimulation response of insecticides on female mosquitoes, regardless of whether they were irritated or spatially repelled by the insecticide. Our results align with those reported by Choi et al. [47], who observed that transfluthrin-exposed mosquitoes were more attracted to oviposition sites, resulting in increased egg-laying, than were non-exposed mosquitoes. Similarly, Rigby et al. [30] reported a 26% increase in eggs per female and a 37% increase in male mating success in resistant *Ae. aegypti* following exposure to sublethal doses of permethrin. These increases were partly attributed to changes in the mosquitoes’ blood avidity and host-locating ability, indicating a behavioral shift following exposure to sublethal permethrin doses [30]. Bong et al. [48] conducted a study where *Ae. aegypti* female mosquitoes were exposed to oviposition sites containing chlorpyrifos. The researchers observed that chlorpyrifos-exposed mosquitoes laid significantly more eggs than did control mosquitoes, indicating the stimulatory effects of sublethal insecticide doses on egg production.

Transgenerational hormesis is common in insects [49]. This phenomenon refers to the effect where the exposure of one parental generation of insects to a sublethal dose of an insecticide results in beneficial effects or enhancements in its progenies. This transgenerational hormetic effect has been observed in various aspects of insect offspring, including growth [50], survival [51–53], body size [51], lifespan [52], and reproduction [54–57]. In the present study, we did not observe any differences in the rate of egg hatching in the progeny of the treated female mosquitoes. However, the egg hatching and development of immature mosquitoes were accelerated. This is probably the first evidence of transgenerational hormesis in mosquitoes.

Another study investigated the effects of sublethal exposure to thiamethoxam on both the parental generation (F0) and the subsequent progeny generation (F1) of *Aphis gossypii* Glover. The researchers [58] reported that when adult aphids in the F0 generation were exposed to a sublethal dose of thiamethoxam (LC_15_), the resulting F1 progeny exhibited stimulatory effects on the pre-adult stage, longevity, and fertility. Moreover, they observed significant increases in the expression levels of key genes involved in the production of vitellogenin and ecdysone receptors as well as cytochrome P450 enzymes in the F1 generation; these increases resulted from parental exposure to thiamethoxam [58]. The mechanisms underlying the reproduction of female *Aedes* mosquitoes and the growth rate of immature mosquitoes (progenies) remain to be elucidated. The changes in gene expression levels likely play crucial roles in mediating the observed improvements in reproduction and immature mosquito growth. This topic deserves further investigation.

## Conclusions

We demonstrated that *Ae. aegypti* mosquitoes exhibit avoidance behavior when exposed to a mixture of deltamethrin and clothianidin, as evidenced by their high percentage of escape from treated surfaces. This finding suggests that IRS with the insecticide mixture effectively deters *Aedes* mosquitoes from entering residential areas, thus reducing mosquito bites. Although we observed delayed mortality and carryover effects of insecticide exposure on the survival of immature mosquitoes, the rate of application might have been insufficient to effectively kill *Aedes* mosquitoes. This finding is unexpected when compared with the results of previous studies using World Health Organization cone bioassays, which reported relatively high mortality rates within 72 h of exposure to residual spraying with the insecticide mixture [19–23, 59]. Notably, we observed stimulated mosquito reproduction and accelerated transgenerational immature development resulting from sublethal doses of the insecticide mixture. This finding indicates that when female mosquitoes survive exposure, their reproductive output may increase, and immature mosquitoes may develop rapidly. These changes in the life history of mosquitoes can have far-reaching consequences, potentially affecting vector management strategies and increasing disease transmission risks. Our findings serve as cautionary notes, highlighting the need to avoid assuming that the recommended application rate of an insecticide product will be universally effective against all mosquito vectors. Each mosquito species may respond differently to insecticides, and their behavioral and physiological responses to sublethal doses can have substantial implications for mosquito control programs.

## Data Availability

The data supporting the conclusions of this study are provided within the article. All datasets generated and analyzed during this study are available from the corresponding author upon reasonable request.
